# MINDFULNESS-BASED COGNITIVE THERAPY STUDY FOR DEPRESSION AMONG KOREAN SPEAKING OLDER ADULTS

**DOI:** 10.1093/geroni/igae098.0139

**Published:** 2024-12-31

**Authors:** Jenny Kim, Eun Jeong Lee, Amanda Shallcross, Seon Ah Ahn, Pallavi Visvanathan, Bohye Park, Hwa Young Chae, Linda Ko

**Affiliations:** University of Washington, Seattle, Washington, United States; Asian American Resource and Information Network Inc, Wood-Ridge, New Jersey, United States; Cleveland Clinic, Cleveland, Ohio, United States; Ahn Counseling Center, Westwood, New Jersey, United States; Manhattan Center for Mindfulness-Based Therapy, New York, New York, United States; University of Washington, Seattle, Washington, United States; University of Washington, Seattle, Washington, United States; University of Washington, Seattle, Washington, United States

## Abstract

Korean American (KA) older adults experience higher risk for depression compared to Non-Hispanic whites. Healthy Mind Healthy Living (HMHL) is a single arm, pre/post pilot intervention study to test and evaluate the effect of an abbreviated telephone-based mindfulness-based cognitive therapy (MBCT-brief) protocol to reduce depression and improve sleep quality and stress. MBCT-brief was adapted culturally and linguistically for older KA adults; participants attended one of three MBCT-brief groups (8-10 participants, N=24), delivered virtually over the span of 8 weeks by a licensed bilingual/bicultural (English/Korean) clinical therapist. Survey data were collected at baseline and 9 weeks post intervention and measured depressive symptoms (QIDS-SR16), sleep quality (PROMIS Sleep Disturbance 4a), and stress (Perceived Stress Scale). The differences in the three outcome measures from pre and post were calculated using paired T-tests. We observed significant differences for changes in mean±SD for depressive symptoms and sleep quality. Post MBCT-brief, participants had a significant reduction in depressive symptoms (8.04±4.70) compared to baseline (10.29±4.22, t(23)=2.87, p=0.004). Similarly, participants had a significant improvement in sleep quality post MBCT-brief (51.03±2.82) compared to baseline (53.23±4.14, t(23)=2.53, p=0.009). Participants also had reduction in stress (17.54±4.56) compared to the baseline (17.21±3.99, t(23)=0.28, p=0.39), but it was not statistically significant. These findings indicate that culturally and linguistically adapted MBCT-brief has potential to improve symptoms of depression, sleep quality, and stress among older KA adults with limited English proficiency. Future larger trials may want to explore the efficacy and effectiveness of MBCT-brief on depression, sleep quality, and stress with a control group.

